# Ultrasound-assisted synthesis of strontium-bismuth titanate and its application in environmental remediation^☆^

**DOI:** 10.1016/j.ultsonch.2025.107380

**Published:** 2025-05-07

**Authors:** Ermelinda Falletta, Anna Donnadio, Nikoletta Mila, Niloofar Haghshenas, Vincenzo Fabbrizio, Riccardo Vivani, Alessia Giordana, Gabriele Perna, Francesco Cottone, Alessandro Di Michele, Claudia L. Bianchi

**Affiliations:** a Department of Chemistry, University of Milan, via Golgi 19, 20133 Milan, Italy; b Consorzio Interuniversitario Nazionale per la Scienza e Tecnologia dei Materiali (INSTM), Via Giusti 9, 50121 Firenze, Italy; c Department of Pharmaceutical Science, University of Perugia, via del Liceo 1, 06123 Perugia, Italy; d Department of Chemistry, University of Turin, via Pietro Giuria 7, Torino 10121, Italy; e Department of Physics and Geology, University of Perugia, via A. Pascoli, 06123 Perugia, Italy

**Keywords:** Ultrasounds, Bismuth, Strontium titanate, Photocatalysis, Piezoelectricity, Remediation

## Abstract

The pollution of water and air in our world has become a global challenge that requires innovative and sustainable strategies. In this work, the ultrasound-assisted synthesis of bare strontium titanate (STO) and bismuth-doped STO (Bi-STO) is proposed to enhance the photocatalytic and piezocatalytic performance of STO in environmental remediation. The materials were fully characterized using various physicochemical techniques, such as XRD, XPS, BET, UV-DRS, PL, SEM, TEM, and piezoelectric measurements, to assess their structural, optical, morphological and piezoelectric properties. The results revealed that Bi-STO induced lattice distortion, reduced band gap energy, enhanced charge separation efficiency, and improved piezoelectricity respect STO.

The activity of the as-prepared materials was investigated in the field of wastewater remediation through three different approaches: photocatalysis, piezocatalysis, and piezo-photocatalysis, applied in the ibuprofen (IBU) degradation, as well as in the photocatalytic removal of volatile organic compounds (VOCs) and nitrogen oxides (NOx) under different irradiation conditions. The results demonstrated that Bi-STO exhibited superior catalytic performance respect STO, achieving up to 60% IBU degradation under piezo-photocatalysis, thanks to the synergy between light and ultrasound. Furthermore, Bi-STO enabled the complete removal of ethanol and NOx and more than 90% propionic acid reduction under UV irradiation.

This study demonstrates the potential of Bi-STO as an efficient catalyst for environmental remediation, offering an alternative to conventional materials. The present work opens new possibilities for optimizing and improving wastewater treatment and air purification.

## 1 Introduction

The progressive deterioration of air, water, and soil quality poses a severe threat to both human health and biodiversity. At the same time, the preservation of ecosystems and biodiversity plays a fundamental role in maintaining and enhancing environmental quality, ultimately benefiting human well-being. Among the most critical challenges, freshwater contamination has emerged as a pressing global issue, driven by population growth, expanding anthropogenic activities, intensive agriculture and livestock farming, and climate change. The World Economic Forum has consistently ranked the degradation of freshwater resources among the top ten major global risks over the past decade [1].

To address water pollution, various remediation strategies have been proposed, among which photocatalysis represents a promising, clean, and sustainable alternative [2]. Photocatalysis operates through the irradiation of a semiconductor catalyst with an appropriate light source (UV, visible, or solar) possessing energy equal to or greater than the band gap energy (Eg) of the material. This process leads to the generation of photoinduced electron-hole pairs, which subsequently participate in redox reactions, forming highly reactive radical species (O_2_•^−^ and •OH). These radicals interact with organic pollutants in solution, breaking chemical bonds and, ideally, leading to their complete mineralization [3]. Therefore, developing highly efficient photocatalytic materials is essential to ensure effective pollutants degradation in diverse environmental scenarios. Furthermore, harnessing natural solar energy not only enhances sustainability but also reduces the operational costs associated with the process.

Perovskites constitute an outstanding class of materials with exceptional physicochemical, photonic, and piezoelectric properties, enabling their application across various fields, including solar energy conversion, electronics, environmental remediation, CO_2_ valorization, and energy storage [4]. Their distinctive crystal structure, denoted as ABX_3_, consists of two different cations (A and B) and an anion (X) [5]. Typically, perovskites refer to perovskite oxides with the general formula ABO_3_. While pristine perovskites exhibit remarkable features, their practical applicability is often hindered by inherent limitations such as low surface area and rapid charge carriers recombination [6]. Consequently, extensive research has been dedicated to enhancing the performance of perovskite-based photocatalysts, with particular emphasis on tailoring their composition and structural properties [7].

Among perovskites, strontium titanate (SrTiO_3_, STO) is a widely investigated cubic perovskite oxide with a band gap energy of 3.0–3.2 eV [8,9], making it active primarily under UV irradiation [8,9]. To extend its applicability to visible light, doping strategies have been explored to modulate its electronic structure [5,10].

STO is traditionally classified as a cubic perovskite oxide and, due to its centrosymmetric crystal structure, is generally considered non-piezoelectric. However, recent studies have revealed that STO can exhibit unexpected piezoelectric behavior under specific conditions. This intriguing phenomenon has been attributed to various factors, including lattice distortions, doping-induced asymmetries, and engineered nanostructures. For example, Rh-doped SrTiO_3_ inverse opals have demonstrated measurable piezoelectric responses, as confirmed by piezoresponse force microscopy (PFM), despite retaining a cubic perovskite phase according to X-ray diffraction (XRD) analysis [11]. This indicates that local structural distortions or internal electric fields introduced through doping and morphological engineering can induce non-centrosymmetric regions within an otherwise symmetric bulk material. Similarly, doping STO with elements such as Sn^4+^ has been shown to provide structural distortions, enhance electrical conductivity and activate local piezoelectric responses while maintaining a nominally cubic structure [12]. In another study, Fe-doped, nonstoichiometric STO ceramics exhibited room-temperature ferroelectric and piezoelectric-like behavior, attributed to lattice distortions, antisite defects, and the coexistence of cubic and tetragonal phases [13]. Kholkin et al. further supported this idea by demonstrating room-temperature surface piezoelectricity in SrTiO_3_ ceramic pellets synthesized via the conventional solid-state reaction method, attributing the behavior to the flexoelectric effect [14] Based on this, Singh *et al.* studied SrTiO_3_ ceramic pellets subjected to ultrasound vibration and proposed that local surface piezoelectricity, along with other ultrasound-induced phenomena, could account for the observed piezo-catalyst activity [15]. These findings suggest that even nominally cubic STO can host nanoscale regions with broken inversion symmetry, enabling local piezoelectric effects.

In addition, recent studies have demonstrated that doping STO with noble and non-noble metals significantly enhances its visible-light absorption and piezo-photocatalytic activity. For instance, Zhou *et al.* reported Rh-doped STO as an efficient photocatalyst for bisphenol A degradation under visible light [16], while Hou *et al.* proposed Cr-doped STO for the photodegradation of azo dyes [17]. Moreover, Liu and colleagues successfully applied similar materials for water splitting [18]. More recently, Bi-modified STO materials have been synthesized and tested for dye degradation, exhibiting promising results [19].

Various physical and chemical approaches, such as hydrothermal and solvothermal methods, ball milling, magnetron sputtering, chemical vapor deposition, solid-state reactions, sol–gel processes, photoreduction, and spray pyrolysis, have been widely employed for the synthesis of diverse nanostructured materials [20]. Each of these approaches offers specific advantages and limitations. However, many of these conventional synthesis methods are costly, labor-intensive, and energy-demanding, often requiring extended processing times. To enhance photocatalytic performance while minimizing energy consumption and processing duration, the development of more efficient synthesis strategies is essential [[21], [22], [23]].

Among the emerging methods, sonochemical synthesis has gained attention as a modern and energy-efficient technique for fabricating materials with outstanding activity [24,25].

In the synthesis of advanced photocatalysts, ultrasound-assisted methods have gained considerable attention due to their ability to produce materials with unique properties [26,27]. The effectiveness of ultrasound stems from acoustic cavitation, which involves the formation, growth, and implosive collapse of bubbles in a liquid medium. These processes generate extreme localized conditions, including transient temperatures of 5000–25000 K, pressures up to 1800 atm, and rapid heating and cooling rates exceeding 10^1^⁰ K s^−1^ [28]. Such conditions facilitate the formation of highly reactive species and significantly influence material crystallinity, morphology, and defect engineering, leading to improved photocatalytic performances.

In this context, ultrasound-assisted methods for titanate synthesis have been partially explored. However, most studies have primarily focused on investigating the relationship between synthesis conditions, morphology, and physicochemical properties [29,30], while relatively little attention has been given to the photocatalytic and piezocatalytic properties of the final materials.

Beyond their well-established photonic properties, many perovskites, such as BaSrTiO_3_ [31], exhibit remarkable piezoelectric behavior. The piezoelectric effect refers to the generation of an electric potential when a piezoelectric material undergoes mechanical stress [32]. This effect has been increasingly explored in photocatalysis, as it provides an efficient strategy for suppressing charge carrier recombination and enhancing charge separation and migration. Despite the potential advantages, reports on the piezoelectric-assisted photocatalytic properties of doped-STO and its derivatives for emerging pollutant degradation remain scarce.

In this study, we present an innovative approach for the ultrasound-assisted synthesis of both pristine and Bi-doped STO. The synthesized materials were thoroughly characterized and evaluated for their photocatalytic performance in the degradation of nitrogen oxides (NOx), volatile organic compounds (VOCs), such as propionic acid and ethanol, and persistent organic pollutants (POPs), such as ibuprofen (IBU), under light irradiation. Notably, Bi-STO demonstrated remarkable piezo-photocatalytic properties during IBU degradation, particularly when exposed to simultaneous ultrasound and light irradiation, and permitted to completely degrade NOx and VOCs under UV light. These findings highlight the potential of ultrasound in the doping of STO with a non-noble metal (bismuth). The doped materials, obtained with highly purity, exhibited high visible light harvesting and piezoelectric features, positively promoting its photo- and piezo-photocatalytic properties.

## 2 Experimental

### 2.1 Chemicals

Strontium acetate (Sr(CH_3_CO_2_)_2_, > 99.5 %), titanium (IV) butoxide (Ti(C_4_H_9_O)_4_, >98 %), bismuth (III) nitrate pentahydrate (Bi(NO_3_)_3_·5H_2_O, ≥ 98.5 %), citric acid (C_6_H_8_O_7_, ≥ 99.5 %), ethanol absolute (C_2_H_6_O, ≥ 99.5 %), ethylene glycol (C_2_H_6_O_2_, ≥ 99 %), ibuprofen sodium salt (C_13_H_17_NaO_2_), propionic acid (C_3_H_6_O_2_, ≥ 99.5) were purchased from Sigma Aldrich and used without further purification. Ultra-pure water was obtained by Milli-Q® ultra-pure water system from Merck Millipore. NOx tank used for photodegradation tests contained 0.625 % of NO_2_ and 0.125 % of NO, diluted with air was purchased by Sapio.

For characterization analyses, liquid nitrogen (N_2_, > 99.0 %, Sapio), barium sulphate (BaSO_4_, >99.0 %, Carlo Erba), nitric acid (HNO_3_, 65.0 %, Carlo Erba), sodium hydroxide (NaOH, >99 %, Carlo Erba), sodium nitrate (NaNO_3_, >99 %, Sigma Aldrich), sodium sulphate (Na_2_SO_4_, ≥99 %, Sigma Aldrich), acetonitrile (CH_3_CN, HPLC grade, Carlo Erba), water (H_2_O, HPLC grade, Carlo Erba), formic acid (HCOOH, 98 %, Sigma-Aldrich), were used.

### 2.2 Materials synthesis

STO and Bi-STO have been synthesized combining hydrothermal and sonochemitry syntheses [[33], [34], [35]], in particular: titanium butoxide Ti(C_4_H_9_O)_4_ and strontium acetate Sr (CH_3_COO)_2_ were used as precursors of Ti and Sr respectively for the synthesis of STO and the corresponding Bi-doped-derivative. More in detail, for the preparation of 5.00 g of STO, 9.2 mL (26.97 mmol) of titanium butoxide were dissolved in 100 mL of ethanol, followed by the addition of 100 mL of ethylene glycol and 3.86 g (20.09 mmol) of citric acid. Finally, 50 mL of an aqueous solution containing 5.55 g (26.97 mmol) of strontium acetate were added dropwise under ultrasound irradiation for 30 min at 80 °C with 50 % amplitude, and 200 W power. The ultrasound generator is an Ultrasonic Porcessor, VC750 (Sonics and Materials, Inc., Newton, CT, USA) 20 kHz with a diameter tip of 13 mm. The obtained suspension was centrifuged and washed several times with water and then dried at 80 °C. The recovered solids were calcined at 500 °C in a furnace for 10 h and subsequently at 850 °C for 2 h.

A similar procedure was applied for the preparation of Bi-STO (nominal composition: Bi_0.02_Sr_0.98_TiO_3_) with slight modification related to the addition of the corresponding doping agent.

For comparison, the synthesis of both the materials was repeated without ultrasound application (STO No US and Bi-STO No US) and the samples were characterized as reported below.

### 2.3 Materials characterizations

Materials crystal structure and phase composition were determined by X-ray powder diffraction (XRD). XRD patterns were collected with a Bruker D8 Advance diffractometer (Bruker AXS GmbH, Karlsruhe, Germany) equipped with a Lynxeye XE-T fast detector and CuKa radiation. The operative conditions were 40 kV and 40 mA, with a step size of 0.033° 2θ and a step scan time of 30 s. Phase identification was performed using the Bruker DIFFRAC.EVA V5 software, and the COD database. Quantitative analyses and structural and microstructural parameters of crystalline phases were performed by applying the Rietveld method using the Bruker AXS Topas Version 6 software. Accurate refined values of lattice parameters were obtained by adding a weighted amount of crystalline silicon powder Si (a = 5.4309 Å) (10 % w/w) to the sample as line position standard. X-ray photoelectron spectroscopy (XPS) analyses were performed by an X-ray photoelectron spectroscope PHI 5000 Versaprobe II, ULVAC-PHI (Inc., Kanagawa, Japan), that employs a monochromatic Al Kα radiation (hν = 1486.6 eV). The binding energies (B.E.) accuracy was ± 0.2 eV. Peak fitting was performed using Gaussian-Lorentzian profiles, with background correction applied via the Shirley method. Binding energy calibration was done by the C1s peak at 284.6 eV.

The zero-point charge (ZPC) of all the materials were determined according to the literature [36]. In 20 mL of NaNO_3_ 0.1 M solutions 50 mg of sample powder were introduced and maintained under stirring. Consequently, the initial pH values (pH_initial_) of the solutions were adjusted between 4–10 by adding appropriate quantities of 0.1 M HNO_3_ or NaOH and the suspensions were maintained under stirring (250 rpm) for 24 h. Then, the suspensions were centrifuged (3000 rpm for 10 min), and the final pH values (pH_final_) were measured. To determine the ZPC, the difference between the pH_final_ and pH_initial_ (ΔpH) is plotted as a function of the pH_initial_ and the intersection of the resulting line where ΔpH = 0 provides the ZPC.

N_2_ physisorption at 77 k was employed for determining the specific surface area of the synthesized samples, a Coulter SA3100 instrument was used. The samples were pre-treated at 150 °C for 4 h in nitrogen flux (Airliquide Alphagaz). Then, the analyses were performed under dynamic vacuum by a rotary pump Edwards E2M1-5. Linearization of the adsorption isotherms was obtained according to the Brunauer – Emmett – Teller (BET) equation (2- parameters 0.05 < p/p°< 0.35), allowing to determine the specific surface area. The desorption branch of the isotherm was employed to determine the pore size and pore volume using the Barret–Joyner Halenda (BJH) method in the 0.3 – 0.95p/p° window.

The optical properties of the synthesized photocatalysts were evaluated through UV–vis diffuse reflectance spectroscopy (UV–vis DRS). The spectra were collected at room temperature in 200–800 nm intervals using a double-beam UV–vis-near-infrared (UV–vis-NIR) scanning spectrophotometer (PerkinElmer Lambda 750 s UV–vis spectrophotometer, PerkinElmer, Waltham, MA) equipped with an integrating sphere assembly. The powder samples were finely ground, uniformly pressed in a circular disk (external diameter ca. 4 cm) and included in the sample holder. The latter was inserted in a special quartz cuvette and placed on the window of the integrating sphere for reflectance measurement. The spectra were measured using BaSO_4_ as a reference. The measured reflectance values (R%) were converted to absorbance (Abs, a.u.) following equation (1):

(1) $$Abs\;=\;log\;(\frac{1}{R}*100)$$

Tauc plot was used to evaluate the band gap energy by equation (2):

(2) (αh)1/2=A(hν-Eg)

where α, h, and ν are the absorption coefficient, Planck constant, and frequency of light, respectively, and Eg is the band gap energy value.

The photoluminescence properties (PL) of the materials were measured at room temperature on the solid samples using a Varian Cary Eclipse fluorescence spectrophotometer, exciting at 320 nm, and 350 nm (slit ex = 20 nm, em = 20 nm).

The morphology and composition of the synthesized materials were examined by Field Emission Gun Electron Scanning Microscopy (FE-SEM) LEO 1525 ZEISS. Elemental composition and chemical mapping were determined using a Bruker Quantax EDX system equipped with a Peltier-cooled BRUKER XFlash 410-M silicon drift detector. Semi-quantitative (standardless) results were based on a peak-to-background ZAF evaluation method (P/B-ZAF correction technology) and a series fit deconvolution model provided by the Esprit 1.9 software (Bruker). EDX analyses were performed at 20 kV on different areas of 15 mm^2^.

While Trasmission Electron Microscopy (TEM) images were obtained using a Philips 208 Transmission Electron Microscope. The samples were prepared by placing a drop of an ethanol dispersion of each powder on a copper grid pre-coated with a Formvar film and air-dried.

**Fig. 1:**
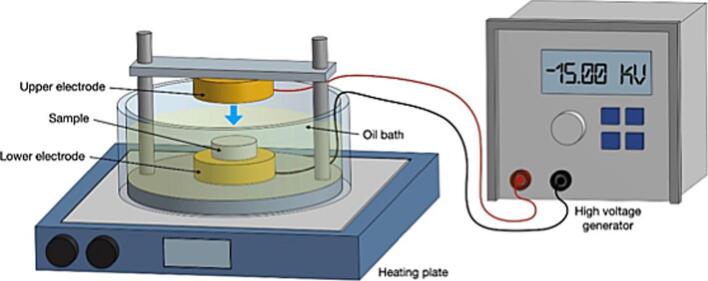
Graphical representation of the experimental polarization setup.

For measuring the piezoelectric properties, the two powders were pressed into disks (0.5 mm of thickness) under a pressure of 1 Ton/cm^2^ in a mold which has a diameter of 12 mm. Successively, as shown in Fig. 1, the disks were polarized between two aluminum electrodes in a silicon oil bath at 100 °C placed on a heated plate, by applying an electric field of 15 kV/mm using the contact method:

the high voltage and ground electrodes were placed on upper and lower surfaces of the sample, sandwiching it. Once the oil bath temperature was set, the generator voltage was activated.

A temperature of 100 °C is maintained for 10 min, after which the heating was switched off, but the applied electric field was kept active until the material cooled down to room temperature, for a total estimated polarization time of 1 h and 30 min. This procedure ensures that the oriented domains within the material generated by the external poling do not de-align, leaving with a remnant polarization. In addition, a suitable poling temperature can rotate the electric domains more easily, which can both increase the piezoelectricity and maintain the polarization for a long time. Afterwards, all poling samples were allowed to set for 24 h prior to measuring the piezoelectric properties. The piezoelectric d_33_ coefficient, that represents the induced polarization in the transversal direction (z axis) per unit stress applied in the same direction, was measured using a Berlincourt piezometer (YE2730A, Sinocera Piezotronics, Inc., China), by applying a periodic force up to an amplitude of 0.25 N with a frequency of 110 Hz on the disk surfaces through the two instrument electrodes made of steel. Electrochemical Impedance Spectroscopy (EIS) and Mott-Schottky (MS) analysis were carried out on an electrochemical workstation (Autolab PGSTAT204, Methrom) coupled with a standard three-electrode system, consisting of a platinum foil (10 x 10 mm^2^) as counter electrode, a saturated calomel electrode (SCE) as reference, and a working electrode prepared as reported in literature [37]. In detail, 10 mg of each material was dispersed in 400 µL of acetone forming a homogenous suspension. Subsequently, 20 µL of the prepared suspension was deposited on a slice of Indium Tin Oxide (ITO) glass (5 x 5 mm^2^) and dried at 25 °C overnight. A 0.1 M Na_2_SO_4_ aqueous solution was used as the electrolytic solution. EIS measurements were carried out both in the dark and under simulated solar light (power density = 35 W/m^2^) to evaluate the photoresponse of the materials, whereas MS investigations were performed in dark.

### 2.4 Photocatalytic tests

#### 2.4.1 Ibuprofen photodegradation in water matrix

Ibuprofen (IBU) degradation was investigated in different conditions: in the dark (dark), under ultrasound application (piezo-), under solar light irradiation (photo-), and under both ultrasound application and solar light irradiation (piezophoto-). All the tests were carried out both in the presence and in the absence of catalyst to evaluate the effect of photolysis, sonolysis and sonophotolysis on pollutant degradation.

All the tests were performed under the same conditions. More in detail, a 250 mL jacketed batch glass reactor was used to maintain the reaction mixture at constant temperature (24.0 ± 0.1 °C) under atmospheric pressure. The reactor was placed in a dark-wall box capable of blocking any external light. A solar lamp was installed on the side of the reactor. The radiation source was an ULTRA VITALUX 300 W-OSRAM solar lamp with power density of irradiation of 35 W/m^2^. A 20 kHz ultrasonic processor (VibraCell VCX 500, Sonics and Materials) equipped with a 13 mm tip composed of a titanium alloy (Ti-6Al-4 V) was placed over the reactor and the US probe (136 mm) was immersed into the aqueous mixture. A pulsed sonication mode was adopted (5 s On/5 s Off) and calibrated as reported by Meroni *et al*. [38]. The ultrasound output power was 23 W.

The tests were executed by stirring (250 rpm) a suspension of 0.25 g/L of catalyst powder in 100 mL of a 50 mg/L IBU solution in ultrapure (MilliQ) water. For all the tests, during the initial 30 min the reaction mixture was kept under dark conditions to permit the achievement of adsorption/desorption equilibrium between the powder sample and the target molecule in solution. Subsequently, solar light irradiation, pulsed sonication or both were turned on for 180 min.

For the monitoring of the pollutant abatement, 1.5 mL aliquots at frequent time intervals were taken. These samples were then placed in 2 mL conical vials and centrifuged with a LaboGene ScanSpeed centrifuge at 13500 rpm for 5 min to separate the catalyst powder before being quantitatively analyzed by a HPLC-UV (Agilent 1100 Series), equipped with a C18 Supelco column (25 cm × 4 mm, 5 μm), a 20 μL injection loop, and a UV–vis detector (Agilent 1100 DAD G1315B). A mobile phase composed of 50 % water and 50 % acetonitrile with an addition of 0.1 % formic acid was used for isocratic elution at a flowrate of 1 mL/min. The IBU concentration decrease was monitored at 230 nm, while the appearance of potential transformation products (TPs) at both 230 and 260 nm.

The pollutant abatement (%) was calculated according to equation (3):

(3) $$abatement\left(\%\right)=\frac{C_{0}-C_{t}}{C_{0}}\cdot 100\%$$

where C_0_ is the initial pollutant concentration, and C_t_ is the pollutant concentration at the time t.

#### 2.4.2 Air pollutants photodegradation

##### 2.4.2.1 Volatile organic compounds (VOCs) photodegradation tests

To evaluate the photoactivity of the synthesized materials towards air pollutants abatement, propionic acid (PA) and ethanol (EtOH) were selected as VOCs model molecules.

50 mg of each photocatalyst were suspended in 15 mL of 2-propanol, sonicated in an ultrasonic bath, and deposited onto an inert glass support (10 × 10 cm^2^) by drop-casting. The coated glass was placed inside a 5.5 L Pyrex reactor containing an initial 500 ± 25 mg/L concentration of each VOC in the vapor phase for the tests performed under UV irradiation and 200 ± 15 mg/L of initial concentration of air pollutants when the tests were carried out under LED. The reactor was placed in a custom-made box dark wall to minimize the external light interferences and inside the reactor a continuous stirring guarantees a certain turbulence of the system. The reactor was kept in the dark for 1 h to permit the achievement of the adsorption/desorption equilibrium between the solid catalyst and the target molecule, then irradiated by the proper light source for 180 min. LED lamp characteristics: 350 mA, 9–48 V DC, 16.8 W with an emission range of 400–700 nm, yielding 2900 LUX intensity on the catalyst surface. UV-A lamp characteristics: HG500, 500 W, 315–400 nm, JELOSIL S.r.L, with a power intensity of 20 W/m^2^ on the catalyst surface. The progress of the reaction was monitored every 30 min by GC/FID analyses, using a Shimadzu GC-2025 gas-chromatograph, equipped with an FID detector (flame ionization detector), equipped with a Teknokroma® column (TRB-625, 30 m 0.32 mm 1.8 μm).

The chromatographic conditions used for the VOCs degradation monitoring were as follows: nitrogen flow rate = 3.00 mL/min, SS inlet = Split mode (split ratio 5:0), injector temperature = 200 °C, detector temperature = 220 °C, nitrogen flow (makeup gas) = 30.0 mL/min, H_2_ flow = 40.0 mL/, air flow = 400.0 mL/min [39].

For the chromatographic elution of the two air pollutants, two oven temperature programs were used, as reported in Table 1.

**Table 1:** GC oven programs.

VOCs	Rate (°C/min)	Temperature (°C)	Hold time (min)
PA	−	90.00	1.00
	70.00	200.00	5.00
	−	50.00	1.00
EtOH	25.00	150.00	3.00
	45.00	180.00	0.00

##### 2.4.2.2 NOx photodegradation tests

All the synthesized photocatalysts were tested for NOx degradation under both LED and UV light irradiation (same lamps used for the VOCs degradation yielding an intensity of 2900 LUX with LED and 10 w/m^2^ with the UV lamp on the catalyst surface). Each sample was dispersed in 6 mL of 2-propanol, deposited by drop-casting on an inert glass support (20 × 2 cm^2^), and placed in a 20 L Pyrex glass cylindrical reactor. The reactor was filled in with NO_2_ that partially decomposes into NO until the chemical equilibrium is obtained, with an initial concentration of 500 ± 30 µg/L and 1000 ± 20 µg/L under LED and UV lamp irradiation, respectively. Inside the reactor a relative humidity between 20–30 % was maintained, whereas a continuous stirring guaranteed a certain turbulence.

The variation of both NO and NO_2_ concentrations during the reaction were instantaneously monitored by an ENVEA AC32e chemiluminescence detector directly connected to the reactor.

## 3 Results and discussion

### 3.1 Chemical-physical investigations of the synthesized perovskites

The application of ultrasonic treatment in synthesis processes plays a crucial role in promoting phase purity and improving the homogeneity of the synthesized materials. In fact, as reported in the literature [20,24], ultrasonic treatment enhances the dispersion and activation of precursors in solution/mixture and reduces local concentration gradients, thereby suppressing the formation of undesired phases. In the present work, SrTiO_3_ (STO) and Bi-doped SrTiO_3_ (Bi-STO) were preliminary synthesized without ultrasonic assistance. If on the one hand STO was obtained as cubic perovskite structure with high purity (X-ray diffractogram reported in Fig. S1a), on the other hand the synthesis of Bi-STO led to a secondary phase (mainly associated to different phases of TiO_2_), as shown in the X-ray diffractogram provided in Fig. S1b. Conversely, the application of ultrasonic energy during the materials’ preparation led to the formation of single-phase perovskite structures, as confirmed below results.

X-Ray diffraction patterns of STO and Bi-STO samples prepared via sonochemical assisted route were very similar to each other (Fig. S2), however, Rietveld refinement procedures were able to highlight small but significant differences.

Both of them were refined with the typical cubic perovskite structure of STO (COD 9006864) under the Pm-3 m space group, while the Bi-STO was found to contain also a very small amount, 2.0(1)% wt/wt, of rutile TiO_2_. Unit cell parameters of the samples were accurately refined thanks to the addition of silicon powder as line position standard. For STO a = 3.90789(2) Å, with a unit cell volume of 59.680(1) Å^3^ while for the Bi-doped sample resulted a = 3.91132(4) Å and a volume of 59.837(2) Å^3^. The slightly larger value of unit cell parameter for the doped sample can be correlated to a partial replacement of Sr with Bi cations. In the STO structure Sr^2+^ ions have a coordination number of 12, and an estimated ionic radius of 1.44 Å, while that of Bi^3+^, extrapolated at this coordination number is 1.45 Å [40].

In agreement to the hypothesis of partial Bi/Sr substitution, and to the Bi/Sr molar ratio of 0.015 found with EDX analyses, in the Rietveld refinement procedure the site occupancy factors of Sr and Bi were set at 0.985 and 0.015, respectively.

Furthermore, XPS analysis showed the presence of Ti^3+^ (see later), that can be justified, for electroneutrality requirements, by the introduction of extra positive charges due to the partial Bi/Sr substitution. In this hypothesis, and assuming that no cationic vacancies or oxygen excess are present, the unit formula of Bi-STO should be as Sr_0.985_Bi_0.015_Ti(IV)_0.985_Ti(III)_0.015_O_3_ (Table S1).

A further information obtained by the Rietveld refinements was the increase of the microstrain value from 0.20(1) % of STO to 0.47(1) % of the Bi-STO sample, while the crystal size remained practically constant (94(2) and 87(2) nm, respectively. This can be interpreted with the increased lattice disorder related to the Bi/Sr substitution.

**Fig. 2:**
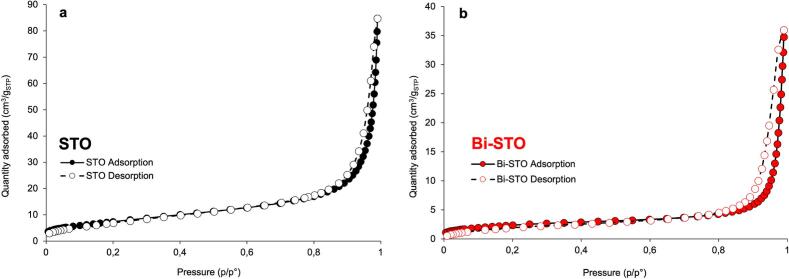
Nitrogen adsorption–desorption isotherms of (a) STO and (b) Bi-STO.

The surface area and porosity of the synthesized perovskites were analyzed through N_2_ adsorption–desorption isotherms collected at −196 °C (Fig. 2). Both materials exhibit type-IV isotherms, according to the IUPAC classification, with a H3 hysteresis loop, which is typical of mesoporous materials [41].

More in detail, STO displayed a surface area of 27 m^2^/g, which is consistent with the value reported in the literature [29,36].

In contrast, the introduction of Bi into the STO lattice resulted in a decrease in surface area to 7 m^2^/g due to a decrease in crystallite size and to an increase of the average microstrain. This resulted in greater aggregation of the nanoparticles as evidenced by TEM analysis (Fig. 9d). These structural modifications are further confirmed by the pore volume values, which also decreased after Bi doping from 0.1325 cm^3^/g to 0.0555 cm^3^/g.

**Fig. 3:**
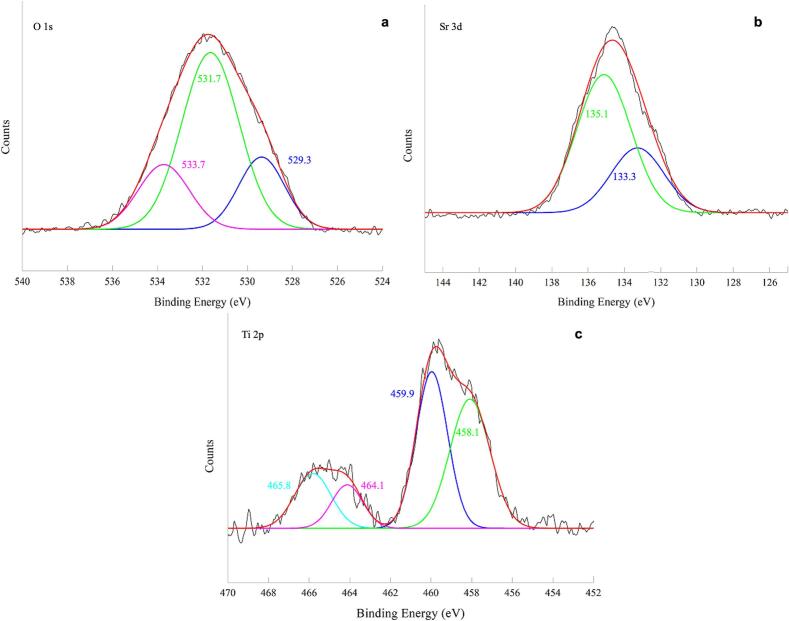
HR XPS spectra of Bi-STO: (a) O 1s, (b) Sr 3d, and (c) Ti 2p regions.

The surface properties of the most performing perovskite, Bi-STO (as described below), were investigated by XPS. The survey spectrum is reported in Fig. S3, whereas Fig. 3 display the high resolution (HR) spectra of Sr, Ti and O.

The survey spectrum (Fig. S3) revealed the presence of C, O, Sr, Bi and Ti as main elements at the material surface.

The HR spectrum of C 1 s (Fig. S4) revealed three main peaks in addition to the adventitious carbon that can be connected to carbonaceous species present on the material's surface. In particular, the pick at 285.8 eV can be connected to C-O species, whereases the pick at 286.9 eV can be attributed to the C=O species [36].

According to the literature [5], in the HR spectrum of Sr3d two peaks are observed at 133.3 eV and 135.1 eV, corresponding to Sr 3d_5_/_2_ and Sr 3d_3_/_2_, respectively.

The three distinct peaks at 529.3 eV, 531.7 eV, and 533.7 eV in the HR spectrum of O1s can be associated to the lattice oxygen, oxygen vacancies, and chemically adsorbed oxygen, respectively [42]. The HR spectrum of Ti 2p can be deconvoluted in four main peaks. The main peaks at 459.9 eV and 465.8 eV correspond to Ti 2p_3_/_2_ and Ti 2p_1_/_2_, respectively, with a spin–orbit splitting of about 5.9 eV, consistent with the Ti^4+^ oxidation state [43,44]. Additionally, other two less intense contributions at 458.1 and 464.1 eV suggest the presence of Ti^3+^ (Ti 2p_3_/_2_ and Ti 2p_1_/_2_). In this case, the spin–orbit splitting for Ti^3^^+^ is approximately 6.0 eV, aligning with previously reported values for Ti^3^^+^ species in perovskite oxides and titanium-based compounds [45]. Finally, the HR spectrum of Bi4f was characterized by peaks of scarce intensity, so no deconvolution in sub-bands was carried out. This evidence confirms the low amount of Bi detected at the material’s surface.

In the field of photocatalytic degradation of pollutants in water matrices, the adsorption of the pollutant on the catalyst’s surface plays a crucial role and represents the first step of the photodegradation process. The zero-point charge (ZPC) is a key parameter that permits to predict the pollutant-catalyst interaction efficiency. When the catalyst is in an environment with pH values below its ZPC, the catalyst’s surface is positively charged, attracting anions, whereas for pH values above the ZPC, the surface is negatively charged, attracting cations. The pH value of 50 mg/L IBU solution was found to be 5.1, and at this pH value IBU is negatively charged (p*K*_a_ of 4.85). For the synthesized materials ZPC values of 7.77 and 7.15 were obtained for STO and Bi-STO, respectively.

Consequently, under the reaction conditions, the IBU adsorption on the catalysts’ surface should be favored.

**Fig. 4:**
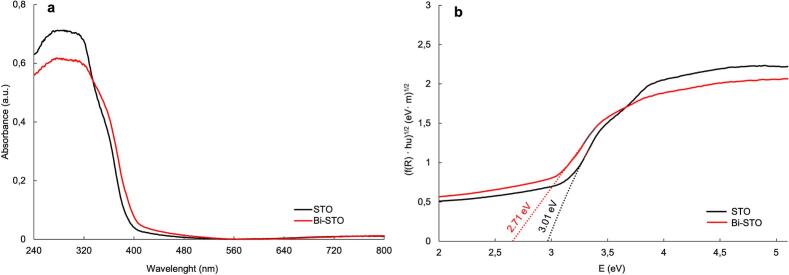
(a) UV–Vis absorption spectra and (b) Tauc plots of STO and Bi-STO, respectively.

The optical properties of the synthetized photocatalysts were evaluated by UV–Vis DRS and the results are displayed in Fig. 4.

As highlighted in Fig. 4a, Bi-STO exhibits a slight red shift in the absorption edge compared to bare STO, indicating an improved absorption efficiency in the visible light region of the electromagnetic spectrum. Furthermore, as shown in Fig. 4b, metal doping reduces the band gap values, demonstrating the effect of the successful incorporation of the metal dopant into the STO structure, as further confirmed by XRD (Fig. S2).

**Fig. 5:**
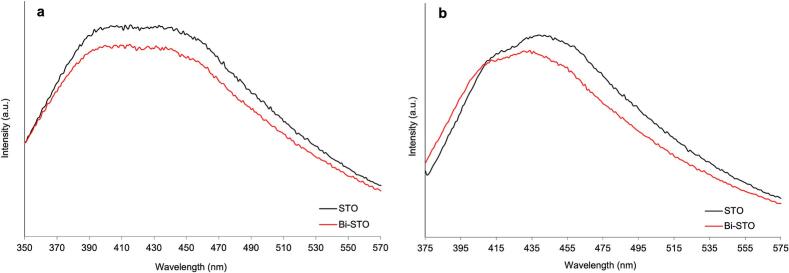
PL spectra of the synthesized photocatalysts at a λ_exc_ = 320 nm (a), and at a λ_exc_ = 350 nm (b).

The photoluminescence (PL) spectra of both the synthesized samples were performed with an excitation wavelength at both 320 nm and 350 nm at room temperature to explore the recombination and/or migration of the e^-^/h^+^ pairs (Fig. 5a and b). Pristine STO exhibits a distinct peak at 435 nm, which can be ascribed to the free excitation movement between band edges, demonstrating the strongest emission peak along with the largest peak area, proving that the photogenerated e^-^/h^+^ are easy to recombine. A decrease in the peak intensity was observed for the Bi-doped perovskite, suggesting that the doping process slightly reduces the photoinduced e^-^/h^+^ charges recombination.

To better understand the electronic properties of the synthesized photocatalysts, Electrochemical Impedance Spectroscopy (EIS) measurement and Mott-Schottky (MS) analyses were performed. EIS was used to evaluate the charge transfer resistance, while MS analysis was conducted to detect any changes in the band structure of STO after doping.

**Fig. 6:**
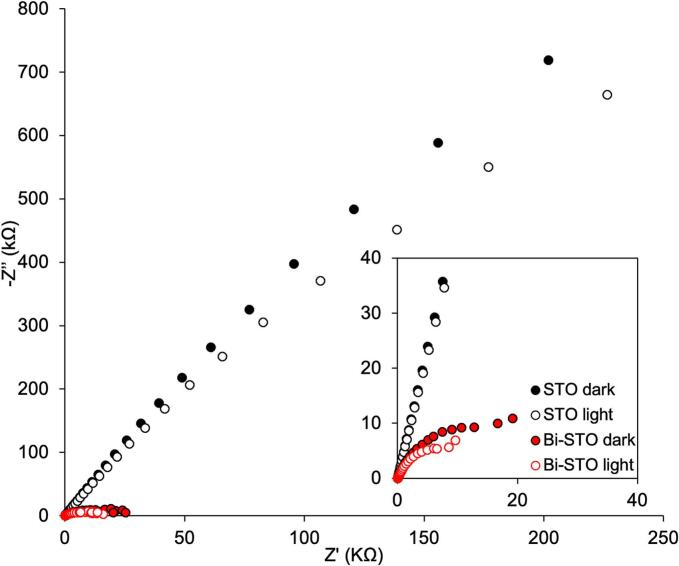
Nyquist plot of STO (black marks) and Bi-STO (red marks) in dark (filled marks) and under simulated solar light irradiation (open marks). (For interpretation of the references to colour in this figure legend, the reader is referred to the web version of this article).

Fig. 6 displays the Nyquist plots of the investigated photocatalysts in dark (filled marks) and under light (open marks).

In Nyquist plot, the radius of the semicircle is indicative of the charge transfer resistance (R_ct_) at the semiconductor–electrolyte interface. In general, a smaller semicircle corresponds to lower R_ct_, which reflects more efficient charge separation and enhanced interfacial charge transport [46]. Bare STO shows a larger arc radius in both conditions, indicating a higher charge transfer resistance and thus less effective separation and transport of photogenerated charge carriers. Conversely, Bi-STO exhibits significantly smaller semicircles, particularly under light irradiation, suggesting that Bi incorporation leads to a notable reduction in charge transfer resistance. Under simulated sunlight, both materials show a decrease in −Z'' values, highlighting improved photo-induced charge separation. However, the effect is more pronounced in Bi-STO, confirming that Bi doping not only enhances conductivity but also facilitates more efficient interfacial charge transfer.

**Fig. 7:**
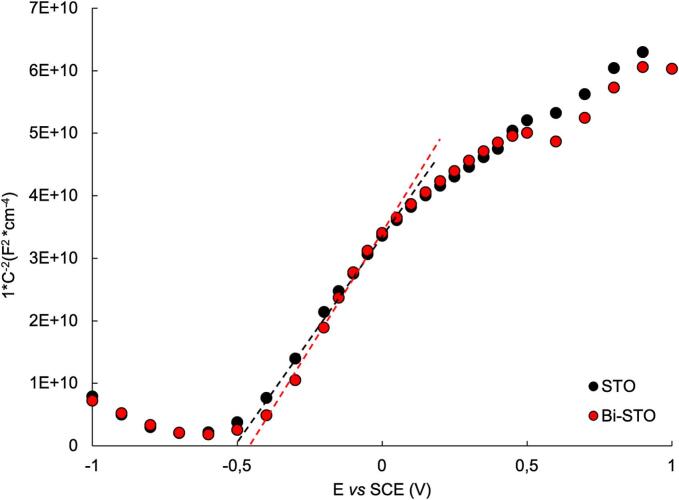
Mott-Schottky plot of STO (black marks) and Bi-STO (red marks). (For interpretation of the references to colour in this figure legend, the reader is referred to the web version of this article).

**Fig. 8:**
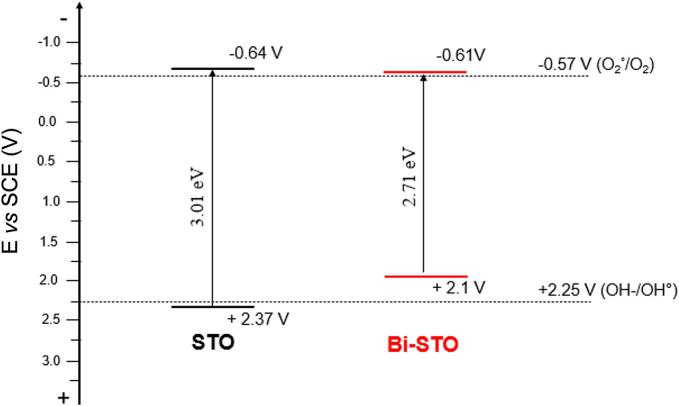
Schematic energy band positions of STO and Bi-STO obtained combining Mott-Schottky analysis and UV–vis DRS results.

Mott-Schottky (MS) plots were used to estimate the flat band potentials (Fig. 7) of the synthesized materials and to determine their band positions (Fig. 8).

As highlighted in Fig. 7, the positive slope of the curves is characteristic of n-type semiconductors [47]. The flat band potentials (E_FB_) were determined by the intersection of the linear part of the plot with the x axis and the E_FB_ values are summarized in Table 2. The flat band potential of bare STO is similar to the values present in literature [48]. The differences in the literature data are due to practical factors, like the method used to prepare the electrode. As highlighted in Table 2, the materials investigated have similar E_FB_ values, suggesting that the metal doping do not affect the conduction band of the as-prepared semiconductors.

**Table 2:** E_FB_ of STO and Bi-STO obtained from Mott-Schottky plot.

Semiconductor	E_FB_ (V) *vs* SCE	Ec_B_ (V) *vs* SCE
STO	−0.54	−0.64
Bi-STO	−0.51	−0.61

Indeed, it is known that for n-type semiconductors, the flat band potential (E_FB_) is typically close to the conduction band edge (E_CB_), within a range of 0.0–0.2 eV more negative [49]. Assuming a 100 mV difference, the E_CB_ of the synthesized semiconductors was estimated. Comparing the E_CB_ estimated from the Mott-Schottky analysis with the bandgap results obtained from UV–vis DRS investigations (Fig. 4b), the E_VB_ of the synthesized semiconductors was estimated (Fig. 8).

It can be observed that, although all the investigated semiconductors have similar E_CB_ values, which can generate O_2_· (E_(O2· / O2)_ = −0.57 V *vs* SCE), metal doping affects the position of the valence band. Specifically, the addition of Bi^3+^ reduces the E_VB_ compared to STO. This shift implies that the doped materials do not have enough energy to generate OH· (E_(HO−/•OH)_ = +2.25 V *vs* SCE), suggesting that the photogenerated holes (h^+^) could be responsible for the pollutants degradation.

**Fig. 9:**
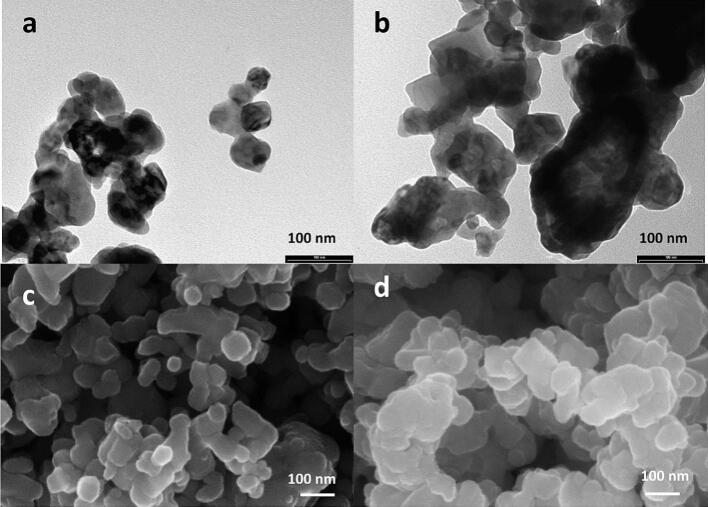
TEM images of STO (a), Bi-STO (b); SEM images of STO (c), Bi-STO (d).

**Fig. 10:**
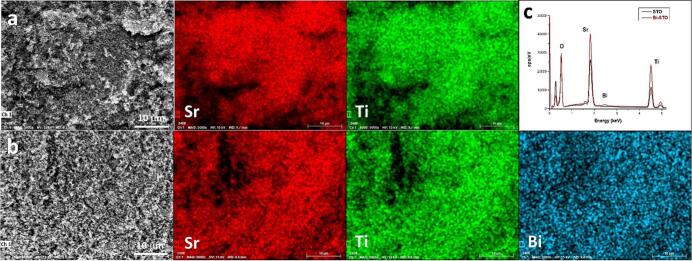
EDX Elemental mapping of STO (a), Bi-STO (b) and EDX spectra (c).

The morphological features of the synthesized samples were investigated by TEM and SEM analyses (Fig. 9), showing similar titanates nanoparticles’ aggregation with well-defined cubic shape and range size between 50 and 90  nm. EDX elemental mapping analyses were done as well, confirming a homogeneous elemental distribution (Fig. 10).

Since the basis of piezoelectric photocatalysis, the piezoelectricity of both STO and Bi-STO was investigated. The obtained d_33_ values of the poled STO samples highlight the effects of Bi doping on STO on the piezoelectric properties for the synthetized materials. The d_33_ value of pristine STO resulted to be very low, 8 pC/N, which confirms the centrosymmetric paraelectric perovskite structure of the material with Pm3m space group. However, this result can explain the piezoelectric-induced catalysis of STO nanocrystals used by Ling et. al. [50] Concerning Bi-STO, the metal doping has the capacity to increase the piezoelectric properties of the material value to 40.9 pC/N, which is 5.1 times that of STO specimen.

### 3.2 Ibuprofen abatement tests

Both the synthesized materials were tested for the abatement of ibuprofen (IBU), used as model molecule for POPs in wastewater. To exploit all the materials’ features, different approaches were employed. Therefore, photocatalytic, piezocatalytic, and coupled photocatalytic and piezocatalytic technologies (piezo-photocatalysis) were properly implemented, as described above.

Initially, with the purpose of verifying the degradation potential of IBU in the absence of any catalyst, tests in the presence of only ultrasound, light irradiation as well as their combination were performed (Fig. S5) and the results indicate an IBU abatement below 10 %. Additionally, 3 h IBU adsorption tests under dark conditions were performed for each catalyst proving that none of the materials exhibit adsorption competitive to photocatalytic features (Fig. S6).

At the beginning of each catalytic test and to achieve the adsorption/desorption equilibrium, the reaction mixture was kept in the dark for 30 min under continuous stirring in the presence of the proper catalyst. Then, the catalytic test was properly performed.

**Fig. 11:**
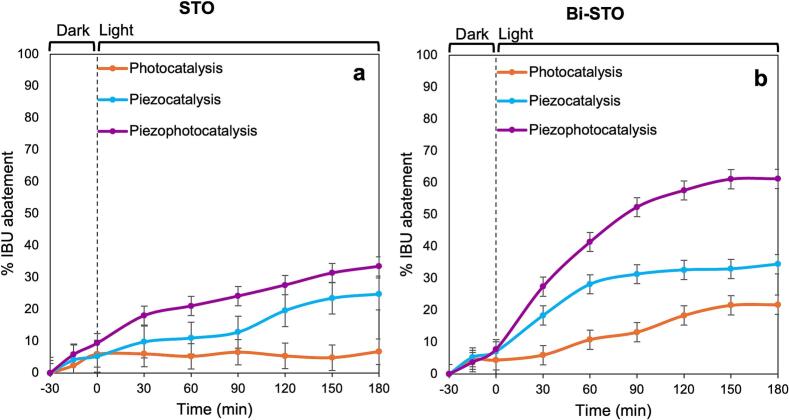
Percentage of IBU abatement by (a) STO and (b) Bi-STO by photo-, piezo-, and piezo-photodegradation processes, respectively.

Fig. 11 displays the results obtained during the IBU abatement by the two single catalytic approaches (photocatalysis and piezocatalysis) and the combined one (piezo-photocatalysis).

Thanks to their piezoelectric features, both the synthesized materials exhibited a piezocatalytic performance higher than the photocatalytic one (Table S2). As expected by the characterization results, the superior piezoelectric d_33_ coefficient along with the increased unit cell distortion of Bi-STO make this latter more performing. Similarly, the reduced bandgap energy and slower recombination rate of the e^-^/h^+^, prolonging their lifetime, improved the sunlight absorption capacity of Bi-STO displaying greater photocatalytic performances, compared to bare STO [51].

When the two techniques (piezo- and photocatalysis) were coupled, piezo-photocatalysis resulted in an improved performance for both catalysts, especially for Bi-STO, rather than when the techniques were applied alone. In fact, the combination of light irradiation with US application enhances the active radical species formation [52,53], improves the mass transfer and regenerates the catalyst surface continuously [54].

The kinetics of the three different approaches (photocatalytic, piezocatalytic and piezo-photocatalytic) were determined by a pseudo-first-order kinetic model. In the plots reported in Fig. S7 the synergistic effect of the combined approach is evident compared to the two single approaches. The evaluation of the kinetic constants (*k*) for each process and each catalyst was used to calculate the synergic factor and the percentage of synergy, according to equations (4), (5) and the results reported in Table 3.

(4) $$\mathrm{synergyfactor}=\frac{k_{\mathrm{piezophotocatalysis}}}{k_{\mathrm{piezocatalysis}}+k_{\mathrm{photocatalysis}}}$$

(5) $$\%synergy=\frac{k_{\mathrm{piezophotocatalysis}}-\left(k_{\mathrm{piezocatalysis}}+k_{\mathrm{photocatalysis}}\right)}{k_{\mathrm{piezocatalysis}}+k_{\mathrm{photocatalysis}}}\cdot 100\%$$

**Table 3:** Kinetic constants of the photocatalytic, piezocatalytic and piezo-photocatalytic IBU abatement, synergy factor and percentage synergy for STO and Bi-STO.

Sample	k_photo_ (min^−1^)	k_piezo_ (min^−1^)	k_piezophoto_ (min^−1^)	Synergy factor	% synergy
STO	0.00005	0.0014	0.0016	1.10	10.34
Bi-STO	0.0013	0.0028	0.0058	1.41	41.46

For both the perovskites a certain percentage of synergy was observed, more pronounced for Bi-STO, in line with its marked optical and piezoelectric features.

In the last decades, efforts have been addressed to the development of efficient photo-, piezo-, and piezo-photocatalysts towards drugs degradation. Table 4 summarizes a few examples.

**Table 4:** Overview of materials used for drugs abatement in literature.

**Catalyst**	**[Pollutant]**	**Light source**	**Ultrasonic vibration**	**Photocatalytic degradation**	**Piezocatalytic degradation**	**Piezo-photocatalytic degradation**	**Ref.**
BiOBr	Ibuprofen 50 mg·L^-1^	Solar lamp, 35 W·m^−2^	20 KHz, 120 W	31 % after 20 min	95 % after 20 min	100 % after 20 min	[55]
ZnO	Diclofenac 25 mg·L^-1^	UV-A lamp, 37 W·m^−2^	20 KHz, 23 W	70 % after 100 min	0 % after 100 min	80 % after 100 min	[56]
Zeolite@TiO_2_	Ibuprofen 300 mg·L^-1^	UV-C, 6 W	Ultrasonic bath	38 % after 100 min	53 % after 100 min	96 % after 100 min	[57]
SnO_2_	Tetracycline 20 mg·L^-1^	LED, 9 W	Ultrasonic bath	55 % after 135 min	45 % after 135 min	88.82 % after 135 min	[58]
N-doped TiO_2_	Ciprofloxacin 40 mg·L^-1^	Blue LED lamp, 14 W	20 KHz	54 % after 210 min	41 % after 90 min	44 % after 90 min	[59]
WO_3_/CNT Carbon nanotube	Tetracycline 20 ppm	Visible lamp, 40 W	24 KHz	45.9 % after 60 min	48.5 % after 60 min	100 % after 60 min	[60]
STO	Ibuprofen 50 mg·L^-1^	Sunlight, 35 W·m^−2^	20 KHz 120 W	10 % after 180 min	25 % after 180 min	30 % after 180 min	This work
Bi-STO	Ibuprofen 50 mg·L^-1^	Sunlight, 35 W·m^−2^	20 KHz 120 W	20 % after 180 min	35 % after 180 min	60 % after 180 min	This work

It is evident that, concerning drugs degradation efficiency, a comparison among different types of catalytic materials is rather challenging. In fact, many parameters affect their performance, such as catalyst, light source, ultrasonic conditions, catalyst dose, pollutant concentration, etc. In addition, very few studies have investigated the piezo-, photo- and synergistic piezo-photocatalytic degradation of IBU. In this regard, BiOBr demonstrated high efficiency in IBU piezo-photocatalytic decomposition, due to its extraordinary piezoelectric properties [55], achieving the complete abatement of the pollutant in just 20 min [55]. In contrast, zeolite@TiO_2_ requires a combination of energetic light (UV-C) and ultrasound application to achieve 96% abatement in 100 min [57]. Although the results obtained by both the synthesized perovskites (STO and Bi-STO) show lower efficiency compared to BiOBr, with a maximum IBU photodegradation of 60%, for the first time the promising potential of perovskites as piezo-photocatalysts for drugs’ abatement is demonstrated. Additionally, the increase in efficiency from STO to Bi-STO indicates a beneficial effect of bismuth incorporation on the interaction between photocatalysis and piezocatalysis. This is especially significant given that their reliance on sunlight makes them more sustainable compared to materials that require only UV radiation. Further optimization of experimental conditions could improve their performance, positioning them as competitive alternatives to the best catalysts documented in the literature.

### 3.3 VOCs photodegradation tests

**Fig. 12:**
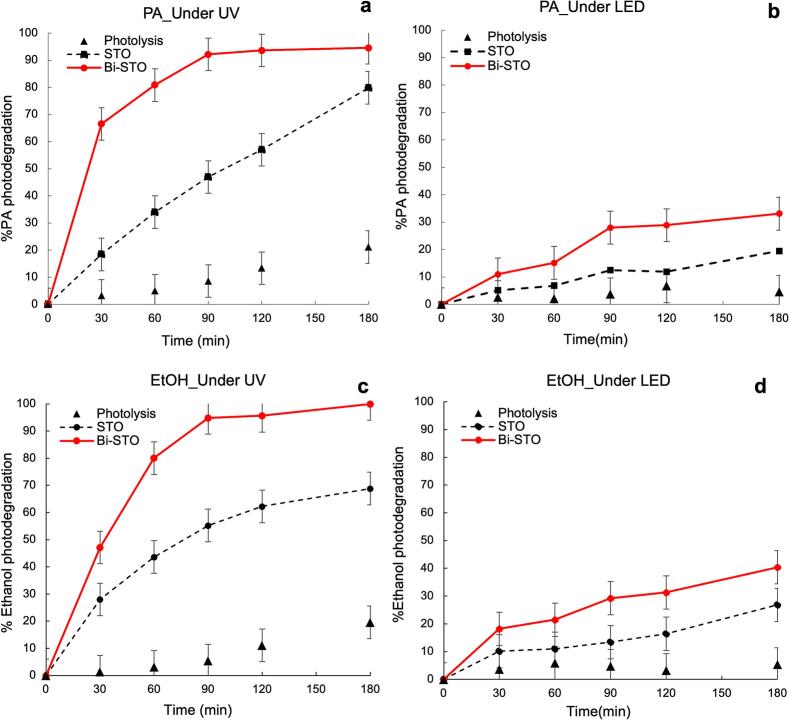
PA and EtOH photodegradation under UV irradiation (a and c) and LED (b and d) by STO and Bi-STO.

Initially, photolysis tests were conducted by irradiating the selected VOCs (PA and EtOH) by both UV and LED light sources. As shown in Fig. 12, approximately 20% of PA and EtOH were decomposed under UV light irradiation in the absence of any catalyst, while no photodegradation occurred when the target molecules were exposed to LED under the same conditions. When the photocatalytic VOCs abatement tests were performed under LED irradiation, the low intensity of the light source caused an energy insufficiency for the initiation of charges separation and the start of photocatalytic processes. However, Bi-STO exhibited slightly higher photoactivity towards both pollutants, reaching 35% and 40% removal of PA and EtOH, respectively, after 3 h irradiation. These results align with the UV–vis DRS analyses in Fig. 4a, where Bi-STO shows a slight red shift in the absorption edge compared to bare STO, suggesting improved absorption efficiency in the visible light region.

In contrast, the synthesized perovskites exhibited outstanding photocatalytic activity when exposed to UV irradiation. Notably, Bi-STO demonstrated excellent photoactivity, achieving nearly 90% PA removal and 100% EtOH removal after 90 min of light exposure, whereas pristine STO achieved a similar final PA removal percentage and 70% EtOH abatement only after 3 h of irradiation.

As discussed above, the positive impact on pollutant degradation is attributed to the bismuth doping effect, preventing the photogenerated electron-hole pairs recombination, leading to a more efficient charge separation, as supported by the photoluminescence analyses (Fig. 5), which show a decrease in the intensity of the Bi-doped photocatalyst's peak, in agreement with the literatures [19,23].

Table 5 provides a selection of both traditional and advanced photocatalysts that have been investigated for the photocatalytic degradation of both PA and EtOH.

**Table 5:** A selective overview of photocatalysts for PA and EtOH photodecomposition.

**VOCs**	**Photocatalyst**	**Percentage of VOC abatement**	**Degradation time (min)**	**Light source**	**Ref**
PA	0.1 %Pt@8%Ag/TiO_2_	60	180	LED Lamp 16.8 W at 0.4mW cm^−2^	[39]
PA	STO	79	180	UV-A lamp HG500, 500 W, at 20 W m^−2^	This work
PA	STO	19	180	LED Lamp 16.8 W at 0.4mW cm^−2^	This work
PA	Bi-STO	92	90	UV-A lamp HG500, 500 W, at 20 W m^−2^	This work
PA	Bi-STO	27	90	LED Lamp 16.8 W at 0.4mW cm^−2^	This work
EtOH	Ag-AgCl/TiO_2_/Cellulose	∼100	60	Solar simulator Xenon lamp (500 W)	[61]
EtOH	TiO_2_ (nano-sized Degussa P25)	100	25	UV-A lamp (500 W) at 30 Wm^−2^	[62]
EtOH	TiO_2_ (micro-sized, 1077 by Kronos)	100	50	UV-A lamp (500 W) at 30 Wm^−2^	[62]
EtOH	TiO_2_ (micro-sized; A-HR by Hunsdman)	100	40	UV-A lamp (500 W) at 30 Wm^−2^	[62]
EtOH	TiO_2_/WO_3_	100	120	UV lamp HG500 17 mW cm^−2^	[63]
EtOH	VOx/TiO_2_ supported on ZnS	76	66 min	UVA-LEDs 90 mW cm^−2^	[64]
EtOH	TiO_2_ films	100	75	UV HG500, 23 mW cm^−2^	[65]
EtOH	TiO_2_ films	100	90	Solar simulator 1 mW cm^−2^ in 280–400 nm 14 mW cm^−2^ in 400–800 nm	[65]
EtOH	STO	68	180	UV-A lamp HG500, 500 W, 20 W m ^-2^	This work
EtOH	STO	26	180	LED Lamp 16.8 W at 0.4mW cm^−2^	This work
EtOH	Bi-STO	100	180	UV-A lamp HG500, 500 W, 20 W m^−2^	This work
EtOH	Bi-STO	40	180	LED Lamp 16.8 W at 0.4mW cm^−2^	This work

As it is possible to observe, identifying the most effective photocatalyst for both PA and EtOH abatement is challenging due to the variability in experimental conditions, such as initial VOC concentration, light source and its effective irradiation power, irradiated area, photocatalyst amount, test duration and so on. All these factors significantly affect the final photoremoval efficiency of each material [66]. However, apart from this, only a few works document the photocatalytic removal of PA in vapour phase, whereas EtOH abatement has been mostly studied by traditional TiO_2_-based catalyst. Therefore, to the best of our knowledge, the results obtained during the UV light-driven photodegradation of both PA and EtOH, by Bi-doped STO, are very interesting and open new perspective into the application of modified-perovskites in this sector.

**Fig. 13:**
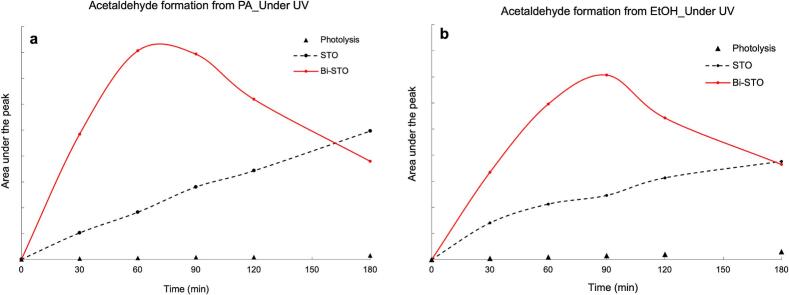
Acetaldehyde trend during the degradation of (a) PA and (b) ethanol with both STO and Bi-STO.

The photodegradation mechanism of both PA and EtOH was already documented in previous works, identifying in acetic acid, ethanol and acetaldehyde the three main transformation products (TPs) [39,67,68]. The evolution of acetaldehyde was confirmed also for the photodegradation processes performed by both STO and Bi-STO, as displayed in Fig. 13, whereas it was not possible to detect the others TPs, probably because produced as traces.

However, it is worth noting that for both PA and EtOH photodegradation in the presence of Bi-STO the degradation of acetaldehyde is very fast if compared to the results obtained by STO.

The acetaldehyde produced in the presence of Bi-STO shows a bell shape trend achieving the maximum concentration level after about 90 min of irradiation, in agreement with the superior catalytic performance of the modified-perovskite. In contrast, the photodegradation processes of both PA and EtOH by STO led to a gradual and progressive accumulation of this TP. Once more, the Bi-doping effect resulted in a power approach also for the abatement of VOCs.

### 3.4 NOx photodegradation

**Fig. 14:**
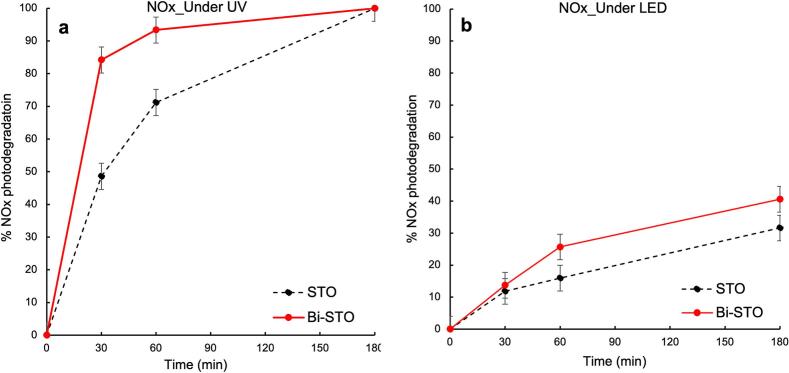
Percentage of NOx photodegradation under UV irradiation (a), and LED (b) by STO and Bi-STO.

Fig. 14 displays the results obtained during the photodegradation tests of NOx by STO and Bi-STO under both UV light and LED irradiation.

As expected on the basis of the results obtained for VOCs abatement, both the perovskites show poor photocatalytic activity when irradiated by LED, whereas their performances increase under UV light irradiation extraordinarily [5].

Also in this case, the STO performance was significantly enhanced by Bi doping, leading to much faster NOx degradation compared to bare STO, achieving over 90 % NOx removal after only after 1 h of UV irradiation. Similarly, than in the previous cases, the beneficial effect of Bi dopant can be attributed to charges separation improvement, lower rate of recombination, and reduced bandgap as confirmed by the characterization results.

Although many efforts have been addressed to the development of innovative performing materials for NOx abatement by light irradiation, to date, most of these still consist of TiO_2_-based photocatalysts, as summarized in Table 6 [69].

**Table 6:** A selective overview of photocatalysts for NOx photodegradation**.**

**Photocatalyst**	**Percentage of NOx abatement**	**Degradation time (min)**	**Light source**	**Ref.**
TiO_2_ nano-sized (Evonik P25)	99	120	UV-A lamp HG500, 500 W 10 W m^−2^	[70]
1 %Ag/TiO_2_	77	180	LED Lamp 16.8 W at 0.4mW cm^−2^	[39]
AgNPs modified- TiO_2_	99	180	LED lamp 350 mA, 9 V 16.8 W, 0.1 mW cm^−2^	[71]
AgNPs decorated- Ag-doped/STO	77	180	LED Lamp 16.8 W at 0.4mW cm^−2^	[72]
AgNPs decorated- STO	75	180	LED Lamp 16.8 W at 0.4mW cm^−2^	[5]
STO	71	60 min	UV-A lamp HG500, 500 W, 20 W m^−2^	This work
STO	31	180 min	LED Lamp 16.8 W at 0.4mW cm^−2^	This work
Bi-STO	87	60 min	UV-A lamp HG500, 500 W, 20 W m^−2^	This work
Bi-STO	40	180 min	LED Lamp 16.8 W at 0.4 mW cm^−2^	This work

Many researchers use NO oxidation to determine the photocatalytic efficiency of materials towards NOx abatement [69,71]. However, NO is converted into NO_2_ in air spontaneously, and this latter is the most toxic of the NOx [73]. Therefore, the data summarized in Table 6 refer to materials employed in the photodegradation of both NO and NO_2_.

Despite the low intensity of the LED source negatively affects the photodegradation efficiency of the synthesized perovskites, particularly Bi-STO, under UV light irradiation the doped STO far exceeds the performance of the corresponding undoped perovskite (STO).

## 4 Conclusion

In this study, the successful fabrication of Bi-STO via ultrasound-assisted synthesis was proposed, highlighting its improved photocatalytic and piezocatalytic properties for environmental remediation. Structural characterization confirmed the incorporation of bismuth into the crystal lattice of STO, leading to a reduced band gap energy, improved charge separation, and improved piezoelectric features compared to pristine STO. Bi-STO exhibited promising results in wastewater treatment, achieving up to 60 % ibuprofen degradation under piezo-photocatalytic conditions, significantly higher than STO. This enhanced activity can be attributed to the synergistic effect of light-induced electron excitation and ultrasound-induced mechanical stress, which improved charge separation and promoted reactive species formation. The photocatalytic performance of Bi-STO was also evaluated in air pollution treatment, demonstrating its effectiveness in removing VOCs and NOx. Notably, it achieved complete ethanol and NOx removal and up to 90 % propionic acid degradation under UV irradiation. The promising results obtained by Bi-STO suggest that perovskite-based photocatalysts could play a key role in next-generation environmental remediation technologies. The synthetic approach used for their synthesis remains, however, the most important aspect to impart peculiar properties to the desired materials.

## CRediT authorship contribution statement

**Ermelinda Falletta:** Writing – review & editing, Writing – original draft, Data curation, Conceptualization. **Anna Donnadio:** Writing – review & editing, Writing – original draft, Investigation, Data curation. **Nikoletta Mila:** Investigation, Data curation. **Niloofar Haghshenas:** Validation, Investigation, Data curation. **Vincenzo Fabbrizio:** Investigation, Data curation. **Riccardo Vivani:** Investigation, Data curation. **Alessia Giordana:** Investigation, Data curation. **Gabriele Perna:** Investigation, Data curation. **Francesco Cottone:** Investigation, Data curation. **Alessandro Di Michele:** Investigation, Data curation. **Claudia L. Bianchi:** Writing – review & editing, Validation, Supervision.

## Declaration of competing interest

The authors declare that they have no known competing financial interests or personal relationships that could have appeared to influence the work reported in this paper.
