# Supplementary material for: Ultrasound-assisted synthesis of strontium-bismuth titanate and its application in environmental remediation
Source: Ultrason Sonochem. 2025 May 7;118:107380. doi: 10.1016/j.ultsonch.2025.107380 (PMC12139497; doi:10.1016/j.ultsonch.2025.107380)
Supplement: Supplementary Data 1 [file mmc1.docx]

**Ultrasound-assisted synthesis of strontium-bismuth titanate and its application in environmental remediation**

Ermelinda Falletta,^a,b^ Anna Donnadio,^c^ Nikoletta Mila,^a,b^ Niloofar Haghshenas,^a,b^ Vincenzo Fabbrizio,*^a,b^ Riccardo Vivani,^c^ Alessia Giordana,^d^ Gabriele Perna,^e^ Francesco Cottone,^e^ Alessandro Di Michele,*^e^ Claudia L. Bianchi^a,b^

1. Department of Chemistry, University of Milan, via Golgi 19, 20133, Milan, Italy
2. Consorzio Interuniversitario Nazionale per la Scienza e Tecnologia dei Materiali (INSTM), Via Giusti 9, 50121 Firenze, Italy
3. Department of Pharmaceutical Sciences, University of Perugia, via del Liceo 1, 06123 Perugia, Italy
4. Department of Chemistry, University of Turin, via Pietro Giuria 7, 10121 Torino, Italy
5. Department of Physics and Geology, University of Perugia, via A. Pascoli, 06123 Perugia, Italy

**Table S1.** Structural data and refinement details for **STO** and **Bi-STO***.*

| Compound | **STO** | **Bi-STO** |
| --- | --- | --- |
| Empirical formula | SrTiO_3_ | Sr_0.985_Bi_0.015_TiO_3_ |
| Formula weight | 183.49 | 185.31 |
| Data range/ 2*θ* ∙deg^-1^ | 20 -120 | |
| Step scan increment, 2*θ*/deg | 0.015 | |
| Step scan time/s | 192 | |
| Wavelength/Å | 1.54060 | |
| N. of data points | 6667 | |
| Crystal system | cubic | |
| Space Group | *Pm-*3*m* | |
| *a*/ Å | 3.90789(2) | 3.91132(4) |
| Volume/Å^3^ | 59.680(1) | 59.837(2) |
| *R_p_* ^a^ | 5.62 | 6.36 |
| *R_wp_* ^b^ | 7.83 | 8.75 |

^a^*R_p_* = Σ |*I_o_-I_c_*| / Σ *I_o_*; ^b^*R_wp_* = [Σ *w*(*I_o_-I_c_*)^2^ / Σ *wI_o_^2^*]^1/2^

**Figure S1.** XR diffractograms of STO (a) and Bi-STO (b) synthesized without ultrasound assisted synthesis.


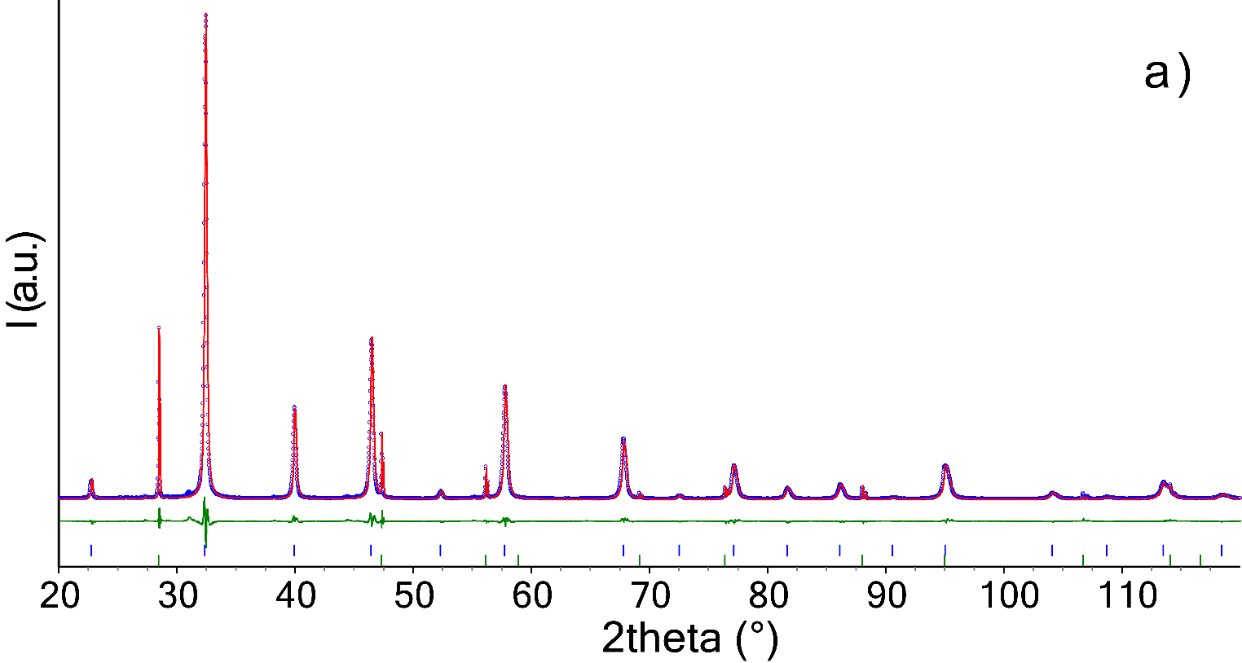


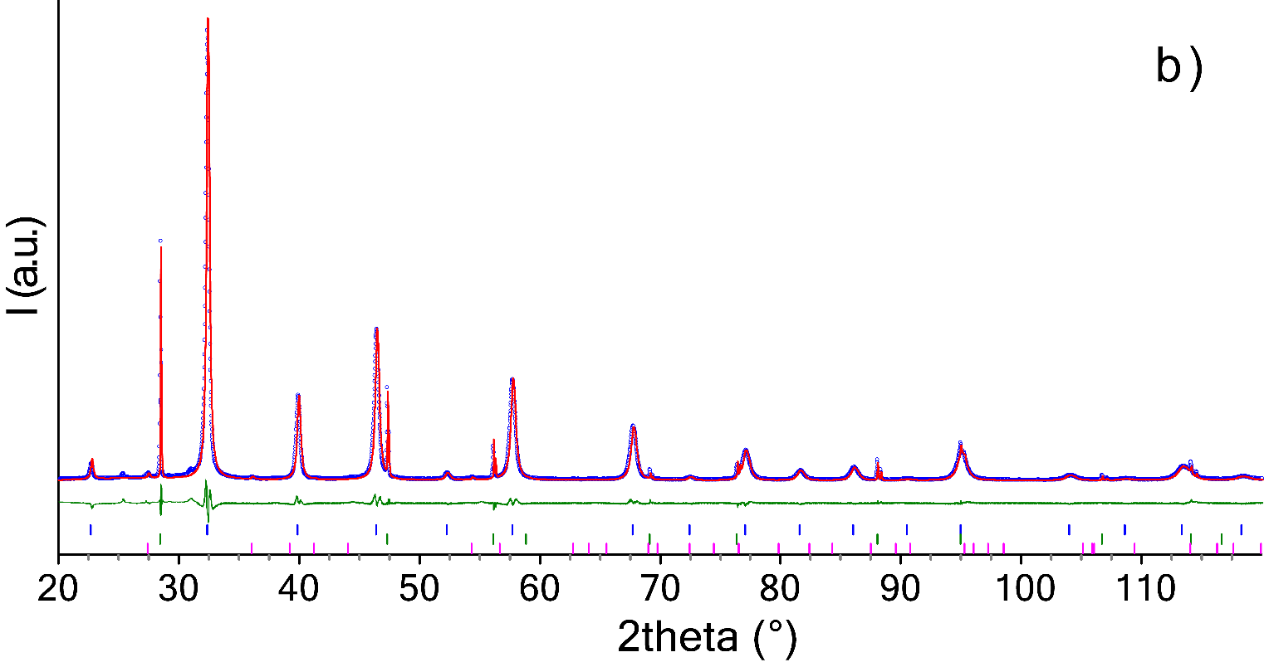


**Figure S2.** Final Rietveld and difference plot for STO (a) and Bi-STO (b). Blue circles: experimental data; red line: calculated profile; green line: their difference. Marks at the bottom indicate the calculated positions of the Bragg peaks of the perovskite (blue), silicon (green), and rutile (pink).

**
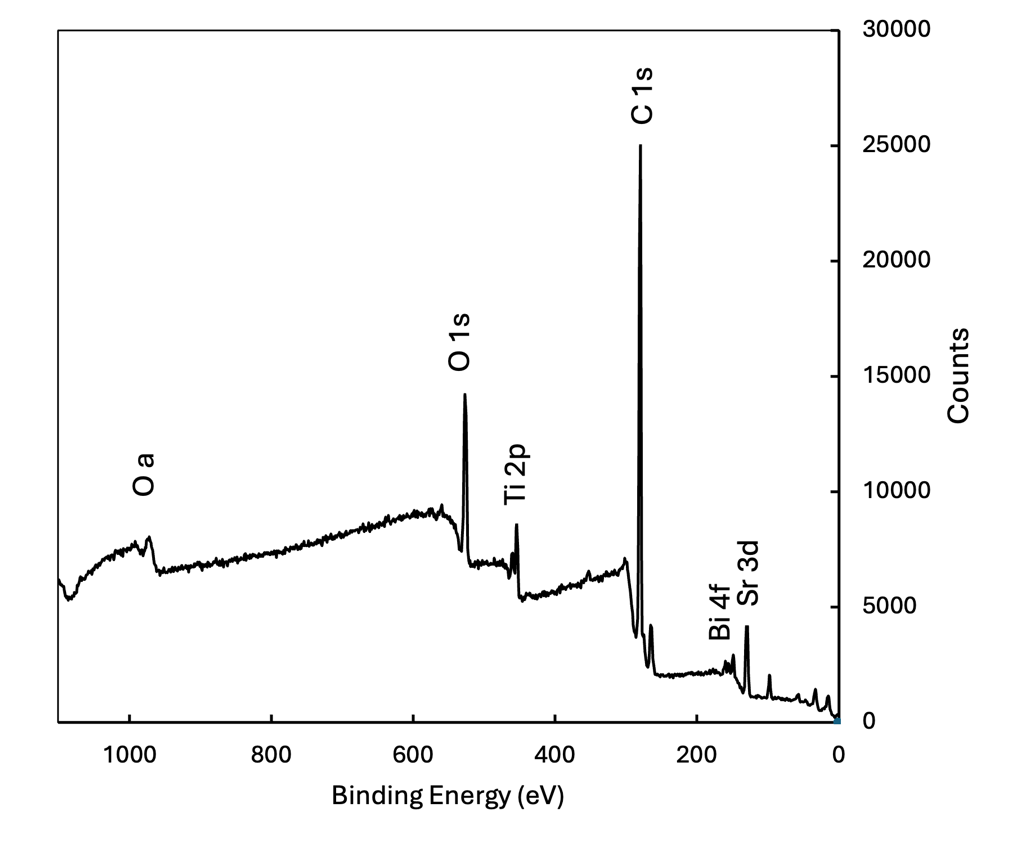
**

**Figure S3:** XPS survey spectrum of Bi-STO.


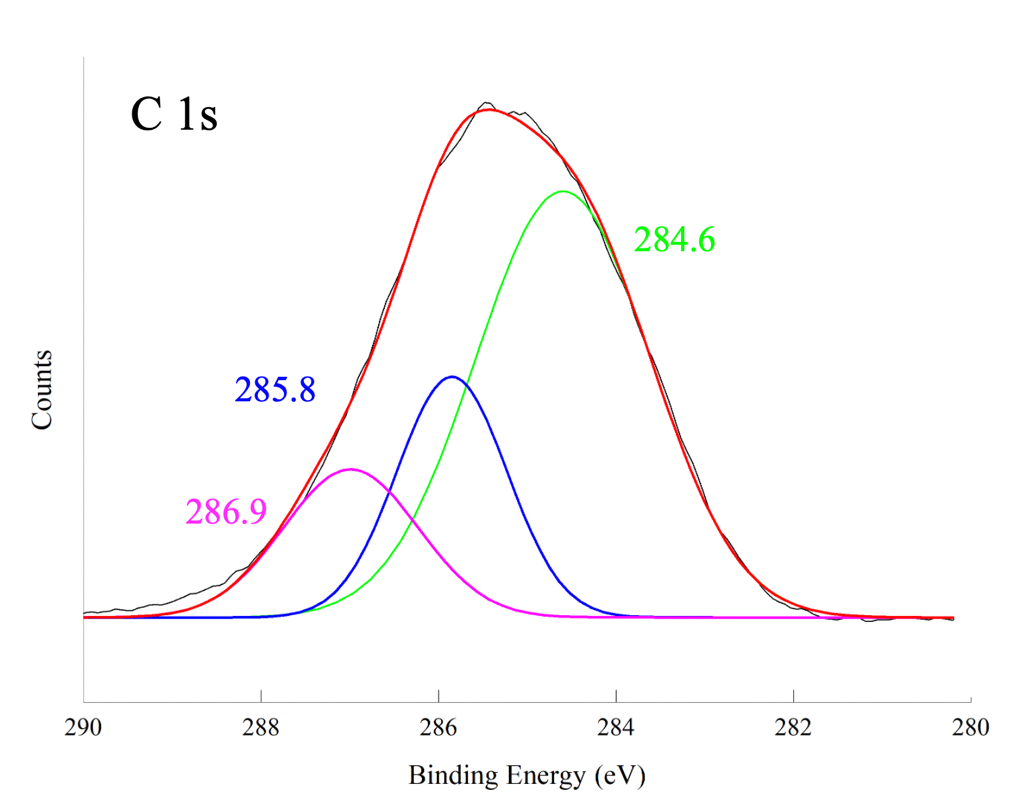


**Figure S4.** HR spectrum of C for Bi-STO.


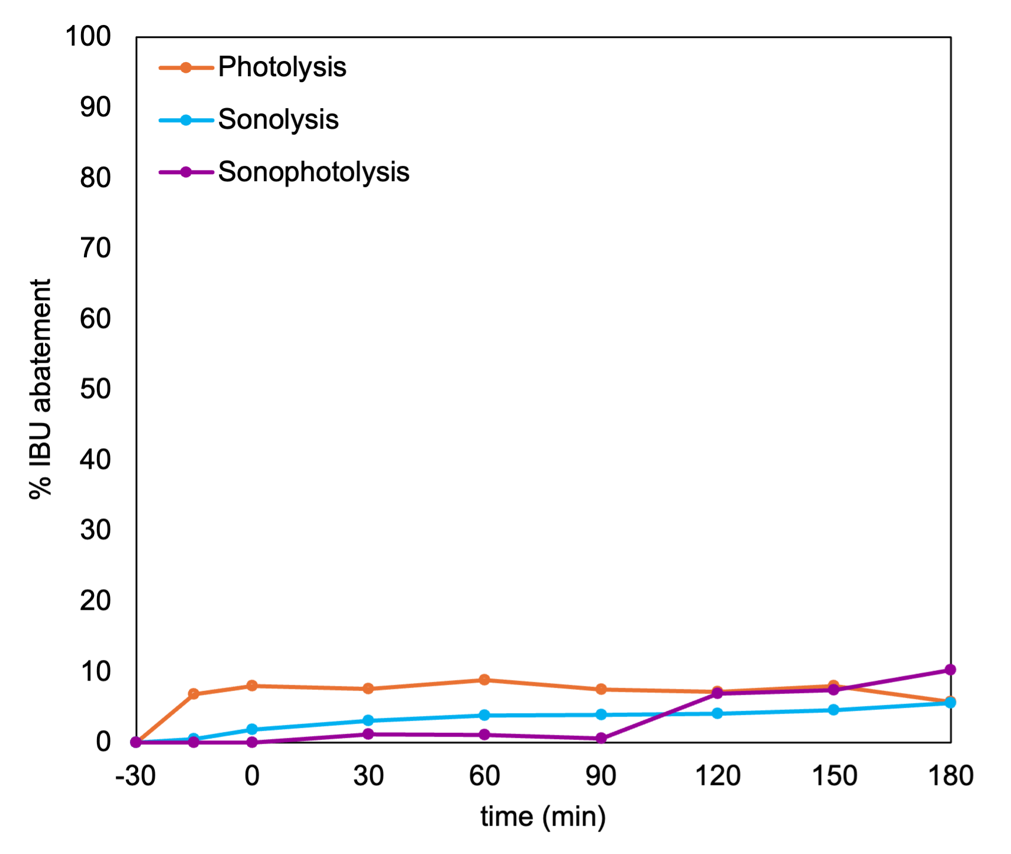


**Figure S5.** Percentage of IBU degradation in the absence of any catalyst.

**
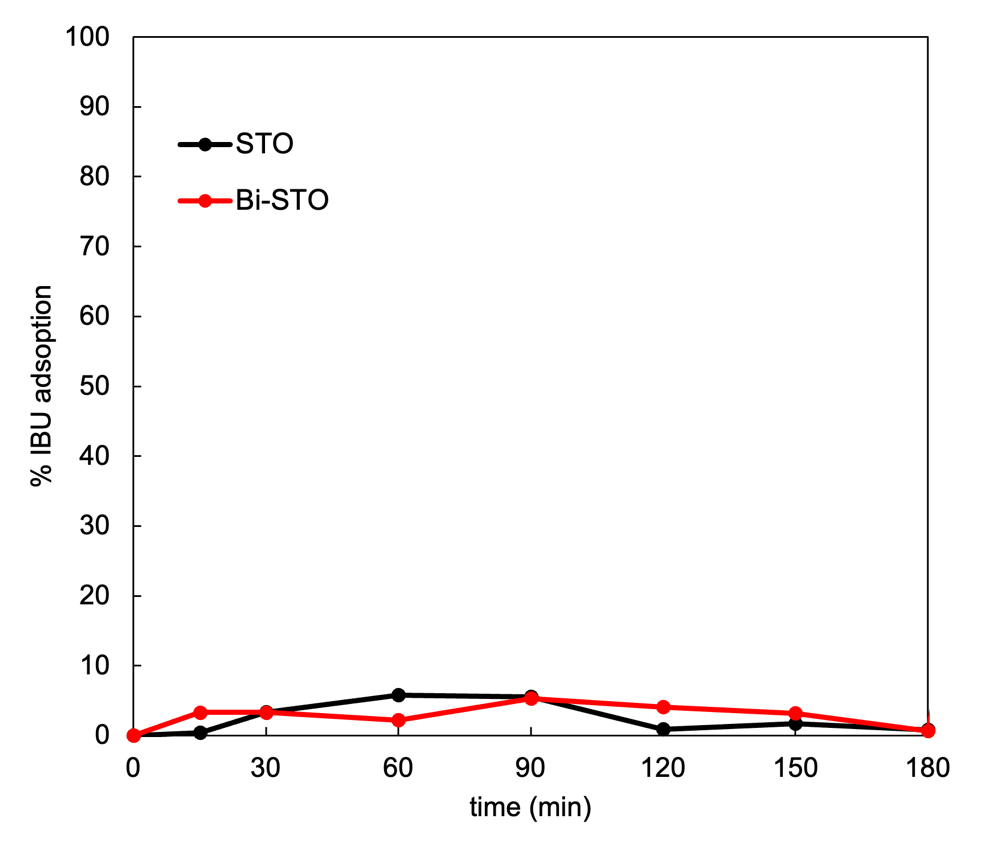
**

**Figure S6.** Percentage of IBU adsorption on STO and doped-STO catalysts.

**
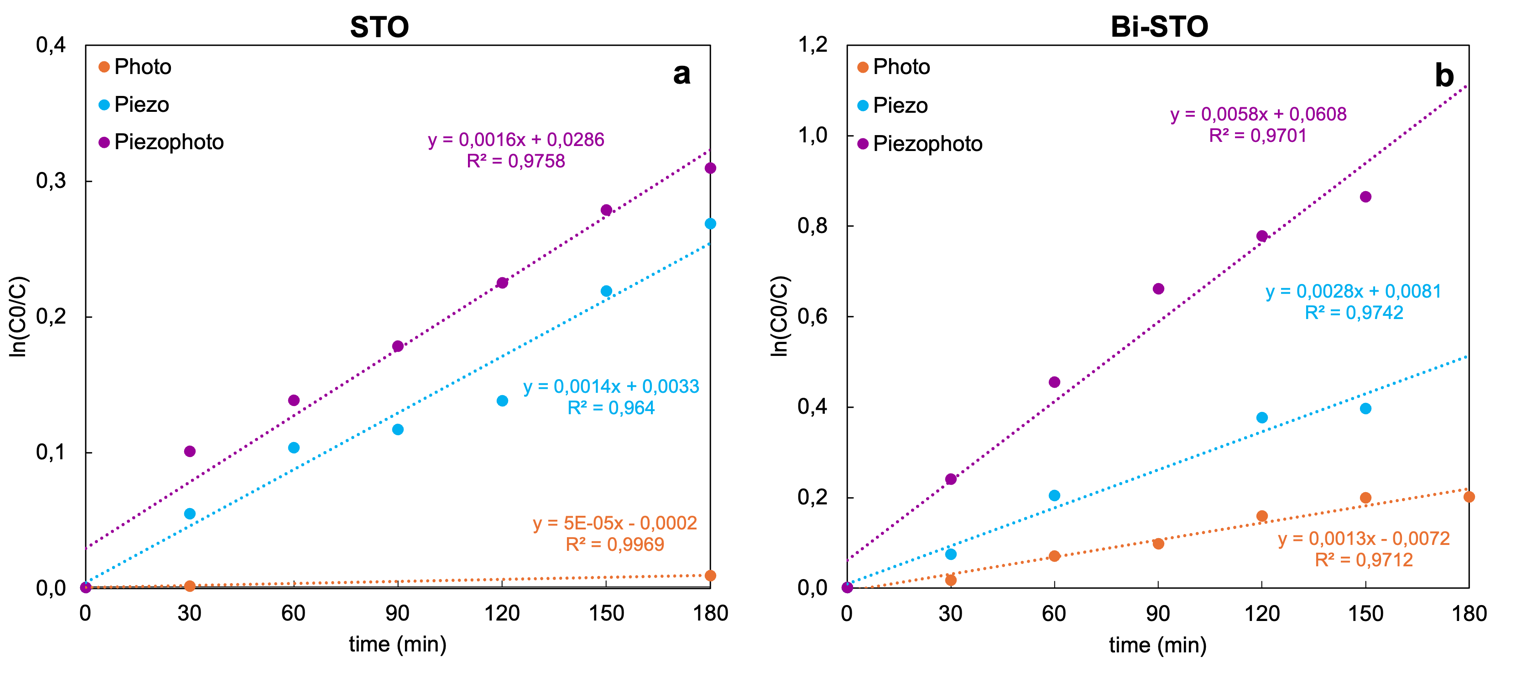

Figure S7.** Linear fittings of pseudo – first – order kinetics of (a) STO and (b)Bi-STO.

**Table S2.** Percentage of IBU abatement results by STO and Bi-STO.

| Catalyst | *Photodegradation* | *Piezodegradation* | *Piezo-photodegradation* |
| --- | --- | --- | --- |
| STO | 6.7 | 24.8 | 33.5 |
| Bi-STO | 21.8 | 34.5 | 61.3 |
